# Developmental Trajectories in Very Preterm Born Children Up to 8 Years: A Longitudinal Cohort Study

**DOI:** 10.3389/fped.2021.672214

**Published:** 2021-05-10

**Authors:** Pauline E. van Beek, Iris E. van der Horst, Josse Wetzer, Anneloes L. van Baar, Brigitte Vugs, Peter Andriessen

**Affiliations:** ^1^Department of Neonatology, Máxima Medical Center, Veldhoven, Netherlands; ^2^Department of Psychology, Máxima Medical Center, Veldhoven, Netherlands; ^3^Department of Child and Adolescent Studies, Utrecht University, Utrecht, Netherlands; ^4^Department of Applied Physics, School of Medical Physics and Engineering, Eindhoven University of Technology, Eindhoven, Netherlands

**Keywords:** longitudinal follow-up, neurodevelopmental outcome, very preterm children, trajectories, NDI

## Abstract

**Aim:** Long-term outcome data in preterm children is often limited to cross-sectional measurement of neurodevelopmental impairment (NDI) at the corrected age of 24-36 months. However, impairments may only become overt during childhood or resolve with time, and individual trajectories in outcome over time may vary. The primary aim of this study was to describe NDI in very preterm born children at three subsequent ages of 2, 5, and 8 years of age. As a secondary aim, a longitudinal analysis was performed on the individual longitudinal trajectories in NDI from 2 to 8 years of age.

**Methods:** Single-center prospective cohort study including children born between 1990 and 2011 below 30 weeks' gestation and followed into 2019. The outcome measurement was NDI assessed at 2, 5, and 8 years of age. NDI is a composite score that includes cognitive, neurological, visual, and auditory functions, in which problems were categorized as none, mild, moderate, or severe. Cognitive function measured as total DQ/IQ score was assessed by standardized psychometric tests. Neurological, visual, and auditory functions were assessed by the neonatologist.

**Results:** In total, 921 children were eligible for follow-up, of whom 726 (79%) children were assessed. No NDI was seen in 54, 54, and 62%, mild NDI was seen in 31, 36, and 30%, and moderate-to-severe NDI was seen in 15, 9.2, and 8.6% of the children at 2, 5, and 8 years, respectively. From 2 to 8 years, 63% of the children remained in the same NDI category, 20% of the children improved to a better NDI category, and 17% deteriorated toward a worse NDI category. No differences were found in baseline characteristics of infants that improved or deteriorated. Extreme prematurity, male gender and low parental education were associated with worse NDI status at all time points. Although we observed considerable individual variation over time in NDI status, the course of the trajectories in NDI were not associated with gestation, gender, and parental education.

**Conclusions:** Continued follow-up until school life is essential in order to provide optimal and individually focused referrals and care when needed.

## Introduction

The number of preterm deliveries below 30 weeks' gestation has increased over the last decades, with increasing survival rates of preterm children (1, 2). However, improved survival rates still raise the concern of adverse long-term outcome in the increasing number of surviving preterm children. Preterm born children are known to have a higher risk of physical disabilities as well as cognitive problems later in life (3–5). Knowledge on neurodevelopmental outcomes of children born at these early gestational ages (GA) is crucial for clinicians and families as this may influence antenatal counseling, resuscitation polices, and NICU guidelines (6–8).

As the impact of developmental impairment may be different at different stages of development, there is increasing interest in studying development as a dynamic process (9, 10). Currently available outcome data are often limited to cross-sectional measurements in toddlerhood, but longitudinal follow-up of children is important. Early suboptimal functioning may form an important signal for later problems or an indication for early intervention, and impairments may persist over childhood into adolescence and adulthood (11–16). Moreover, there may be considerable variation in individual trajectories that is not detectable in cross-sectional studies (17).

Studies evaluating developmental trajectories in preterm children often have focused on specific components of development, like cognitive, behavioral, or social problems (5, 10, 13, 18–20). However, a composite outcome score combining different domains provides a general insight in the amount and kind of disabilities of preterm born children. As developmental problems can arise over a broad spectrum of outcome measures, evaluation of developmental trajectories using a composite outcome might provide additional information. A frequently used indication of adverse long-term outcome is the composite measure of neurodevelopmental impairment (NDI), a score that takes cognitive, neurological, visual, and auditory function into account (8, 21–24). This outcome measure focuses on severe impairments and provides important prognostic information for clinicians and parents (21).

Since three decades, preterm children born below 30 weeks' gestation are eligible for an extensive follow-up program in our perinatal center. This includes outpatient clinic visits to the neonatologist and psychologist at the corrected age of 2 years and the uncorrected age of 5 and 8 years, making an NDI assessment possible at three subsequent ages. The data collected over a period of more than 20 years provides unique information on the development of very preterm children. Therefore, the primary aim of this study was to describe NDI in very preterm born children who were evaluated at three subsequent ages of 2, 5, and 8 years of age. As a secondary aim, a longitudinal analysis was performed on the individual longitudinal trajectories in NDI from 2 to 8 years of age.

## Materials and Methods

### Patient Population

This cohort study included all children born between 1990 and 2011 and followed into 2019, with a gestational age below 30 weeks, who were admitted within 24 h after birth to the neonatal intensive care unit (NICU) of Máxima Medical Centre (MMC). The NICU of MMC serves a 1.6 million population including antenatal and postnatal transfer from six other hospitals in the region. Children from parents living outside the adherence area of MMC and referrals from other NICUs were excluded. The ethical review board of MMC approved the study in accordance with the Dutch law on medical research with humans (WMO).

### Data Collection

Data from the outpatient clinic visits were collected prospectively. Neonatal data were retrieved from the individual medical records. Individual characteristics and medical data included gender (male or female); birth weight in grams; gestational age in days (based on ultrasound findings or on the first day of last menstrual period if ultrasound data was not available); small for gestational age [defined as birth weight below the 10th percentile (25)]; multiplicity (dichotomized as single or multiple birth); mode of delivery (dichotomized as vaginal or by caesarean section); complete course of antenatal corticosteroids (defined as two doses of betamethasone given 24 h apart before the start of labor); Apgar score at 5 min postpartum; inborn or outborn NICU; rate of artificial ventilation > 12 h; days of endotracheal intubation on any mode of ventilation; surgical treatment of a persistent ductus arteriosus; intraventricular hemorrhage grade 3 or 4 based on ultrasound (26); cystic periventricular leukomalacia grade 3 (27); severe brain injury (defined as intraventricular hemorrhage grade 3 or 4 or cystic periventricular leukomalacia grade 3); laparotomy for necrotizing enterocolitis or single intestinal perforation; surgical treatment or laser therapy for retinopathy of prematurity; and total days of NICU admission. Socio-economic status was assessed using scores defined by the Netherlands Institute for Social and Cultural Research (The Hague, Netherlands*)* based on postal code at birth, with an average score of 0 and a positive score reflecting a higher than average status and a negative score reflecting a lower than average status (28). For children not seen for follow-up, reasons for no show were identified.

### Follow-Up

All preterm children below 30 weeks' gestation were eligible for our follow-up program. This consisted of outpatient clinic visits to the neonatologist and psychologist at the corrected age of 2 years and the uncorrected age of 5 and 8 years. The neonatologist assessed the child's health and evaluated the neurological, visual, and auditory functions. Neurological outcome was scored as normal, mildly abnormal, or unilateral/bilateral CP, according to the GMFCS classification (29). The psychologist evaluated the child's cognitive function, emotional, and behavioral development. At the corrected age of 2 years cognitive development was assessed using the Mental Developmental Index of the Bayley Scales of Infant Development-II (BOS2-30 for children born in 1990-2001 or BSID-II, for children born in 2001-2007) or the Cognitive Composite score of the Bayley-III (for children born in 2007-2011). At the age of 5 years cognitive function was tested using the Total IQ score of the Revised Amsterdam Child Intelligence Test short form (RAKIT, for children born in 1990-2008), the Total IQ score of the Revised Amsterdam Child Intelligence Test-2, short form (RAKIT-2, for children born in 2008-2009) or the Total IQ score of the Dutch version of the Wechsler Preschool and Primary Scale of Intelligence Scale-III (WPPSI-III-NL, for children born after 2009). A strong correlation of 0.76 has been reported between the RAKIT and the WPPSI for total IQ scores (30). At the age of 8 years cognitive function was tested using the Total IQ score of the Revised Amsterdam Child Intelligence Test, short form (RAKIT, for children born in 1990-2005), the Total IQ score of the Revised Amsterdam Child Intelligence Test-2, short form (RAKIT-2, for children born in 2005-2006) or the Total IQ score of the Dutch version of the Wechsler Intelligence Scale for Children-III (WISC-III-NL, for children born after 2006). IQ-scores of the RAKIT and WISC have shown a strong correlation of 0.82 (30). In addition, the psychologist collected information on educational status of the parents, which was classified as low, middle, or high according to the CBS classification (31). This variable was dichotomized describing whether there was a low education or middle-to-high education. If one of the parents was classified as middle-to-high educated, parental education was classified as middle-to-high.

### Neurodevelopmental Outcome

The outcome measure was neurodevelopmental impairment (NDI), a composite score based on cognition, neurological assessment, and presence of visual and/or hearing impairment (Table 1). NDI was categorized as none, mild, moderate, or severe. NDI was classified as mild if cognitive scores showed a developmental quotient (DQ) or intelligence quotient (IQ) between 70 and 84 (−2 to −1 SD); vision or hearing loss without an aid or with good correction, or abnormal neurological tests in the absence of a neurological syndrome (e.g., posture, coordination, and tone dysregulation disorders). NDI was scored as moderate if cognitive DQ/IQ scores were between 55 and 69 (−3 to −2 SD); limited vision or hearing and the use of aids or the presence of a unilateral cerebral palsy. NDI was scored as severe if cognitive DQ/IQ scores were below 55 (>-3 SD), or blindness, deafness, or bilateral cerebral palsy were present. NDI score was based on the worst determinant in either one of the four categories. If one category was missing, NDI was classified as missing. NDI was determined for examinations at 2, 5, and 8 years of age.

**Table 1:** Classification of neurodevelopmental impairment.

	**Neurology**	**Vision**	**Hearing**	**Cognition**
No NDI	Normal	Normal	Normal	>-1 SD
Mild NDI	Abnormal neurological tests but absence of neurological syndrome	Vision loss without an aid or with good correction	Hearing loss without an aid or with good correction	−2 to −1 SD
Moderate NDI	Unilateral cerebral palsy	Limited vision and the use of aids	Limited hearing and the use of aids	−3 to −2 SD
Severe NDI	Bilateral cerebral palsy	Blindness	Deafness	< -3 SD

*NDI, neurodevelopmental impairment; SD, standard deviation. Overall NDI score was based on the worst determinant in either one of the four categories*.

### Statistical Analyses

Children with and without follow-up were compared using the Student's *T*-test or Mann-Whitney *U*-test for continuous variables, depending on distribution of the data, and using the Chi-square test for categorical and dichotomous variable. Parental education was missing for 25% of the children and imputed using the R multivariate imputation by chained equation (MICE) package. A continuation ratio model was used to investigate trajectories in NDI, using an interaction term between age and group to test whether NDI trajectories were different for different groups. These group terms included a dichotomized variable for gestational age [extremely preterm (EP) < 28 weeks vs. very preterm (VP) 28^0^-29^6^ weeks' gestation], gender (boys vs. girls), and parental education (low education vs. middle-to-high education). First, the effect of being EP was evaluated by adding this group factor to the model. Then, the effect of gender and parental education were examined by adding them separately to the model. A *p*-value < 0.05 was considered significant. Analyses were performed using R version 3.5.1.

## Results

### Study Population and Loss-to-follow-Up

**Figure 1:**
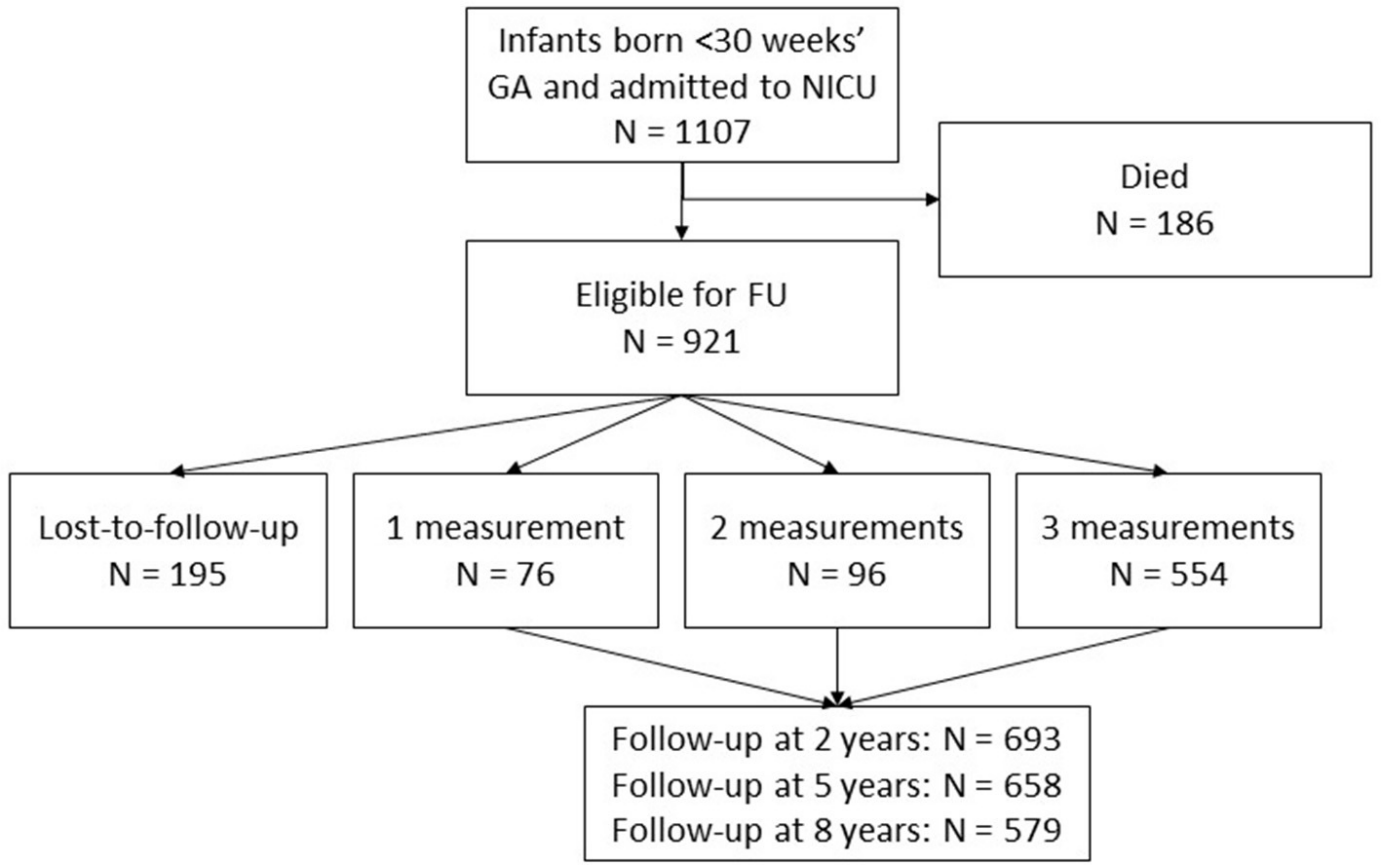
Flowchart of the included children. 921 children were eligible for follow-up at the outpatient clinic. In total, the results are based on 726 participants with data on 1930 follow-up moments, as presented in the gray square.

Within the study period (1990-2011), 1,107 children born below < 30 weeks' GA were admitted to the NICU. Of these children 186 (17%) died, leaving 921 children eligible for follow-up at the outpatient clinic (Figure 1). Of these children, 726 (79%) were seen for follow-up. In total, 693 (75%), 658 (71%), and 579 (63%) children had follow-up at 2, 5, and 8 years, respectively. Reasons for the total group of 195 loss-to-follow-up children are shown in Table 2. During a limited period of time, difficulties in availability of staff resulted in a group of 102 relatively low risk children who were not invited for follow-up.

**Table 2:** Reasons for no follow-up.

**Reason no follow-up**	***N* (%)**
**Logistic reasons**	
Not invited for follow-up due to difficulties in staff	102 (52%)
No show (reason unknown)	34 (17%)
Moved or distance too far	9 (4.6%)
**Parental reasons**	
Parents refused follow-up	11 (5.6%)
No need for follow-up according to parents	6 (3.1%)
**Follow-up elsewhere**	
Ambulatory or clinic for rehabilitation medicine	15 (7.7%)
Follow-up program at other NICU	18 (9.2%)
**Total**	**195 (100%)**

### Baseline Characteristics

Table 3 shows the baseline characteristics, separately for children with and without follow-up. Children with follow-up were more immature at birth, compared to children without follow-up. Socio-economic status was higher in children seen for follow-up. Their NICU admission was significantly more often complicated by PDA and ROP, but less often complicated by a laparotomy. The length of NICU stay in children with follow-up was longer, compared to children without follow-up.

**Table 3:** Baseline characteristics for children with and without follow-up.

	**Children with follow-up**	**Children without follow-up**	***P*-value**
	***N* = 726**	***N* = 195**	
Inborn	672 (93)	175 (90)	0.255
Gender (% male)	394 (54)	104 (54)	0.989
Birth weight	1,037 (259)	1,169 (254)	< 0.001^*^
Gestational age (days)	28.3 [27.9, 29.1]	28.7 [27.9, 29.4]	< 0.001^*^
Gestational age < 28 weeks	310 (43)	52 (27)	< 0.001^*^
SGA (<10th percentile)	90 (12)	7 (3.6)	0.001^*^
Singleton	490 (68)	119 (62)	0.090
Caesarean section	347 (48)	77 (40)	0.061
Antenatal corticosteroids completed	464 (64)	125 (65)	0.231
Apgar 5 min	8 [7, 9]	8 [7, 9]	0.095
Socio-economic status	0.10 (0.82)	−0.07 (0.79)	0.009^*^
Ventilation > 12 h	495 (68)	125 (65)	0.205
Days ventilation	3 [0, 8]	3 [0, 7]	0.241
Surgically treated PDA	51 (7.0)	6 (3.1)	0.007^*^
Severe brain injury	38 (5.3)	11 (5.8)	0.925
Laparotomy	18 (2.5)	14 (7.3)	0.003^*^
Laser therapy for ROP	15 (2.1)	2 (1.0)	0.038^*^
Length of stay in the NICU (days)	36 [22, 48]	22 [13, 40]	< 0.001^*^

*N (%), mean (SD) or median [1st quartile, 3rd quartile]. SGA, small for gestational age; PDA, patent ductus arteriosus; ROP, retinopathy of prematurity; NICU, neonatal intensive care unit*.

* *Significant on a p-level of 0.05*.

### NDI Classification

NDI could be calculated for 646, 618, and 560 children at 2, 5, and 8 years, respectively (Table 4). No NDI was seen in 54, 54, and 62%, and moderate-to-severe NDI was seen in 15, 9.2, and 8.6% of the children at 2, 5, and 8 years, respectively. Of the 201 infants with mild disabilities at 2 years of age, 25 (12.4%) had a mild disability in two domains. None of the infants had disabilities in more than two domains. Of the 225 infants with mild disabilities at 5 years of age, 49 (21.8%) had a mild disability in two domains, 7 (3.1%) had a mild disability in three domains, and 1 (0.4%) had a mild disability in all domains. Of the 167 infants with mild disabilities at 8 years of age, 29 (17.4%) had a mild disability in two domains, 1 (0.6%) had mild disability in three domains, and 1 (0.6%) had a mild disability in all domains.

**Table 4:** NDI at each follow-up age.

**Seen for follow-up**	**Age 2 *N* = 693**	**Age 5 *N* = 658**	**Age 8 *N* = 579**
NDI status unavailable	*N* = 47	*N* = 40	*N* = 19
Included in analysis on NDI	*N* = 646	*N* = 618	*N* = 560
**Age at assessment**			
Mean (SD)	2.28 (0.13)	5.08 (0.19)	8.11 (0.22)
Median (IQR)	2.25 [2.22-2.29]	5.03 [5.01-5.21]	8.03 [8.01-8.23]
**NDI**			
None	351 (54)	336 (54)	345 (62)
Mild	201 (31)	225 (36)	167 (30)
Moderate	46 (7.1)	37 (6.0)	33 (5.9)
Severe	48 (7.4)	20 (3.2)	15 (2.7)
**NDI in EP (<28 weeks)**			
None	144 (50)	117 (47)	129 (55)
Mild	93 (33)	113 (45)	84 (36)
Moderate	26 (9.1)	14 (5.6)	16 (6.8)
Severe	23 (8.0)	6 (2.4)	6 (2.6)
**NDI in VP (28**^**0**^**-29**^**6**^ **weeks)**			
None	207 (58)	208 (61)	212 (68)
Mild	108 (30)	98 (29)	77 (25)
Moderate	20 (5.6)	21 (6.2)	16 (5.1)
Severe	25 (6.9)	12 (3.5)	8 (2.6)

*This table shows NDI rates at 2, 5, and 8 years of age. In the upper half of the table, age at assessment and overall NDI rates are presented for each follow-up age. In the lower half of the table, NDI rates are presented for extremely preterm vs. preterm infants. NDI, neurodevelopmental impairment; SD, standard deviation; IQR, interquartile range; EP, extremely preterm; VP, very preterm; GA, gestational age*.

When they got older, more children were seen in a clinic for rehabilitation medicine and dropped-out on follow-up. Including these children in the category moderate-to-severe NDI, the percentage at 8 years increased up to 16%. In further analysis, the original data was used categorizing this subgroup as missing. Separate presentation of NDI rates for EP and VP infants showed decreased “no NDI” and increased “mild NDI” rates in EP infants compared to VP infants, but similar moderate-to-severe NDI rates (Table 4). In Appendix 1, classifications for the separate components of NDI are presented for each follow-up age.

### NDI From 2 to 8 Years of Age

**Figure 2:**
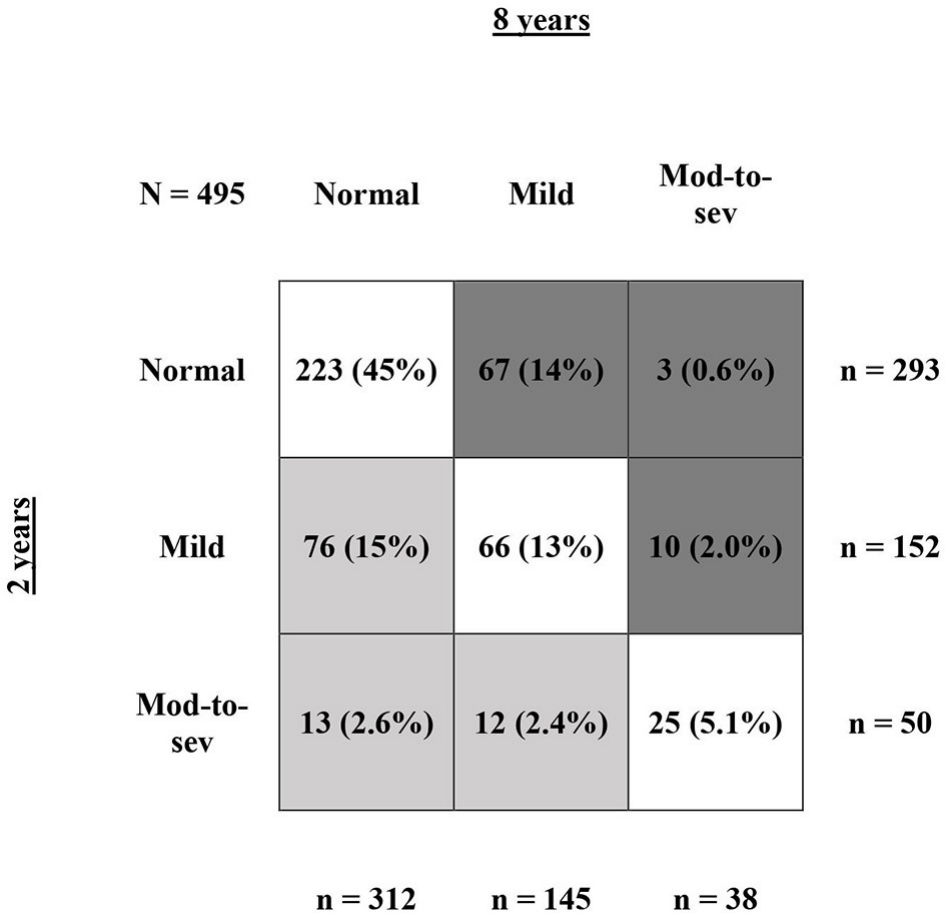
Shifts in NDI from 2 to 8 years of age. This figure shows NDI rate at 2 vs. 8 years of age for infants with NDI calculation at all three follow-up contacts (*N* = 495). The numbers are presented as *N* (%), with the % calculated relatively to the full group of *N* = 495 infants. The row sums show the total number of infants at 2 years of age for normal, mild, and moderate-to-severe NDI. The column sums show the total number of infants at 8 years of age for normal, mild, and moderate-to-severe NDI. The dark gray boxes represent all infants that deteriorated toward a worse NDI category from 2 to 8 years, the light gray boxed represent all infants that improved toward a better NDI category from 2 to 8 years, and the white boxes represent all infants that remained in the same NDI category.

In the 554 children with three follow-up contacts, NDI could be calculated at all time points for 495 children. No NDI during the complete trajectory at 2, 5, and 8 years of age was seen for 179 (36%) children and both no-or-mild NDI during the complete trajectory was seen for 427 (81%) children. Moderate-to-severe NDI during the complete trajectory was seen for 21 (4.2%) children. In these 495 children, from 2 to 8 years 314 (63%) children remained in the same NDI category, 101 (20%) children improved toward a better NDI category, and 80 (17%) children deteriorated toward a worse NDI category (Figure 2). Of all 293 children with normal NDI at 2 years, 223 (76%) remained in the normal NDI category at 8 years of age. For mild impaired infants 43% (66/152) and for moderate-to-severe impaired infants 50% (25/50) remained in the same NDI category. No differences were found in the characteristics of infants that remained in the same category, improved or deteriorated from 2 to 8 years (Table 5).

**Table 5:** Baseline characteristics of children that remained in the same NDI category, improved toward a better NDI category and deteriorated toward a worse NDI category.

	**Children that remained in the same NDI category**	**Children that improved toward a better NDI category**	**Children that deteriorated toward a worse NDI category**	***P*-value**
	***N* = 314**	***N* = 101**	***N* = 80**	
Gender (% male)	163 (52)	53 (53)	37 (46)	0.634
Birth weight	1,039 (249)	1,061 (255)	1,005 (282)	0.339
Gestational age (days)	28.4 [27.1, 29.0]	28.3 [27.1, 29.1]	28.0 [26.9, 29.1]	0.788
SGA (<10th percentile)	34 (11)	9 (8.9)	12 (15)	0.418
Low maternal education	37 (14)	13 (17)	11 (17)	0.738
Severe brain injury	15 (4.8)	3 (3.0)	0 (0.0)	0.116
Length of stay in the NICU (days)	34 [22,48]	36 [23, 45]	35 [23, 48]	0.994

*N (%), mean (SD) or median [1st quartile, 3rd quartile]. SGA, small for gestational age; NICU, neonatal intensive care unit*.

### Individual Longitudinal Trajectories in NDI

**Figure 3:**
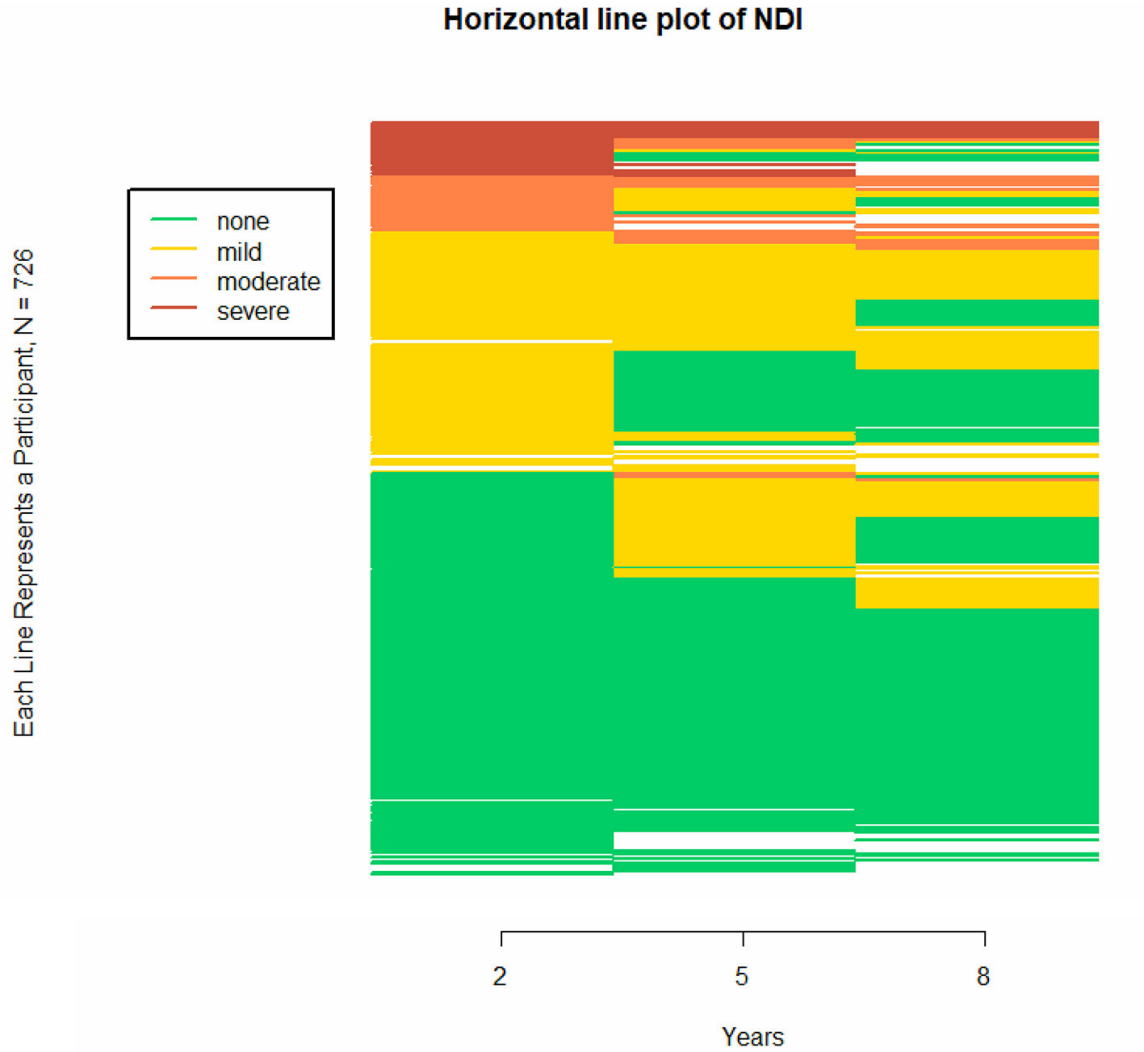
Individual trajectories of neurodevelopmental impairment (NDI) at different ages, presented in a horizontal line plot for all children with follow-up. The horizontal line plot uses colors to differentiate between states on a categorical longitudinal variable for multiple participants. Because categorical data defines a specific state, a trajectory for a categorical variable becomes a sequence of states rather than a continuum. The figure consists of 726 stacked horizontal line, with each horizontal line representing a participant. In this figure, NDI category is presented at 2, 5, and 8 years of age. This figure shows overall patterns of increasing and decreasing trajectories, including patterns in missing data. The blank spaces indicate missing data for that follow-up age.

In clinical work individuals are more important than (sub)groups. Therefore Figure 3 presents the horizontal line plot for NDI at 2, 5, and 8 years of age, showing individual patterns of increasing and decreasing trajectories for all infants including patterns in missing data. Using the continuation ratio model, we found that at all time points very preterm born children had on average a 1.95 (95% CI 1.28-2.96) times higher odds on being in a better NDI category compared to extremely preterm born children. Female children had a 2.00 (95%CI 1.32-3.05) times higher odds compared to male children on being in a better NDI category, and children from parents with middle-to-high education had a 3.37 (95%CI 2.01-5.64) times higher odds compared to children from parents with low education on being in a better NDI category. Studying the trajectories in relation to these characteristics, it was found that EP and VP children showed similar trajectories, as did male and female children and children from parents with low vs. middle-high education.

## Discussion

In this study, neurodevelopmental impairment at 2, 5, and 8 years was evaluated in very and extremely preterm children born below 30 weeks gestation. In addition the course of the individual longitudinal trajectories over time was studied. We observed individual variation over time in NDI status in 37% of the children, with 17% showing a change to a more worrisome category, but 20% showing an improvement. However, 63% of the children remained in the same category over time. Longitudinal analysis showed a clear association of gestation, gender, and parental education with the severity of NDI at all time points. No differences were found in the characteristics between children that improved and deteriorated, and the course of the trajectories in NDI was not affected by gestation, gender, and parental education.

Compared to other studies we observed higher rates for children without or with mild NDI. At the age of 5 and 8 years, respectively, 54 and 62% of the surviving children showed a normal neurodevelopment, and 36 and 30% of the surviving children showed a mild neurodevelopmental impairment. In EP children, normal development rates were 47 and 55%, and mild NDI rates were 45 and 36% at 5 and 8 years, respectively. The Swedish EXPRESS study found rates of 36 and 30% for children without and with mild NDI at 6.5 years in children born below 27 weeks' GA (32). The EPICure study from the UK showed a rate of 75% for children with none-to-mild NDI at 6 years and a rate of 53% for children with none-to-mild NDI in 53% at 11 years, in children born below 26 weeks' GA (3, 33). Unfortunately, international comparisons are hampered by differences in age of follow-up, definition of neurodevelopmental impairment and study population (21). For example, the EPICURE and EXPRESS studies included substantially more immature children, born at 22-24 weeks' gestation, whereas in our sample the youngest children were born at 25 weeks' gestation.

Mild neurodevelopmental problems were seen in 31, 36, and 30% of the infants at 2, 5, and 8 years of age. However, mild deficits in multiple domains might be of the same severity as one moderate-to-severe deficit in a single domain. At 2, 5, and 8 years of age, 12.4, 25.3, and 18.6% of the infants with mild NDI had mild problems in more than one domain. Apparently, at a later age more multiple mild deficits become overt. Multiple deficits across domains may have combined long-term effects, which unfortunately is not reflected by the NDI definition. The significance of milder forms of neurocognitive deficits might need additional research (34).

The moderate-to-severe disability rate in the current study initially appeared to be 8.8% at 8 years of age. However, it was found that 13% of the children lost for follow-up at 8 years of age did not attend follow-up because they were already in treatment in rehabilitation medicine. Including these children as having moderate-to-severe disability resulted in a disability rate of 16%, which is slightly higher than the severe disability rate of 13% reported in both the EPICure and EXPRESS studies (3, 32). On the other hand, children in our study were also lost-to-follow-up because they did not experience any problems. The real moderate-to-severe disability rate probably is somewhere between 8.8 and 16%. This emphasizes the importance of presenting impairment rates in the context of reasons for loss-to-follow-up.

Despite the abundancy of cross-sectional follow-up studies a paucity exists in longitudinal follow-up. This study showed that approximately two third of the children assessed at 2 years of age were classified in the same NDI category at 8 years of age, and that 16% of all children became worse at 8 years of age. Similar results have been reported before in the EXPRESS study, reporting that 47% of all children remained in the similar NDI category and 32% of all children deteriorated toward a worse NDI category from 2 to 6.5 years of age (32). Although overall NDI rates remained comparable over time, these results demonstrate considerable individual variation over time. Indeed this also shows the importance of continuing follow-up until school life for individual and specific referrals and advise.

EP/VP status, gender, and parental education were found to be associated with severity of NDI at all time points. These results were in line with previously published studies, reporting gender-differences in neurodevelopmental outcomes in the favor of girls (35–38). Moreover, these results enhance the formerly reported association between gestational age and neurodevelopmental outcome as well as the association between parental education and neurodevelopmental outcome (11, 36, 38–40).

Although EP/VP status, gender and parental education were found to be associated with NDI, these associations remained stable over time and the course of the trajectories was not affected by these factors. Children with these characteristics therefore seem to have the same developmental growth potential as children without these characteristics (18). Moreover, no differences were found in characteristics between infants that improved or deteriorated from 2 to 8 years. Nevertheless, considerable individual differences were seen in trajectories. This indicates the importance of other factors that might influence development over time, for example early childhood interventions such as an extensive physiotherapy program or special education assistance. Moreover, socio-environmental factors such as the quality of the parent-child relationship are important throughout development (16).

Although extensive evaluation of separate domains is important, the added value of a composite outcome is that it provides an overall impression of the outcomes after very preterm birth. Problems after preterm birth occur in a range of developmental domains and therefore it is important not to focus on single domains of development. Looking separately at the specific domains in this study, the majority of the children did not have any impairment. However, combining the different domains into the NDI composite outcome showed no NDI during the complete trajectory for a minority of 36% of all children. Apparently, the majority of the very preterm children do experience some clear problems at some time during childhood. Moreover, the combined outcome measure used in this study is the longer term outcome most frequently used for comparisons both within and between countries (21). International comparisons can guide clinical decision-making and provide prognostic information for families.

In this study, cognitive scores were corrected for prematurity at age 2, but not at age 5 and 8. The current Dutch national guideline on follow-up and most international guidelines recommends the use of corrected scores for preterm children up to 2–3 years. However, in very preterm children at age 5, a significant difference between corrected and uncorrected IQ was found, with corrected scores of course being higher than uncorrected scores (41). For future research, consistent reporting of cognitive outcome based on corrected scores is recommended (42).

The overall follow-up rate in this study was 79%, which is comparable to follow-up rates of other studies, showing rates varying from 71 to 92% at different ages (3, 5, 32). Moreover, more than 60% of the children completed follow-up at all time points during the longitudinal follow-up program, which demonstrates a high follow-up rate compared to other longitudinal studies such as the recently published EPICure2 study (follow-up rate 19%) (33). Our results might represent the worst-case scenario as medium risk children have not always been invited for follow-up during the study period because of limited resources as shown in Table 2. These children without follow-up were children with an appropriate birth weight, without severe brain injury and with uncomplicated NICU admission. This explains why infants seen for follow-up were more immature at birth and had an increased length of stay in the NICU compared to infants without follow-up. On the other hand, a significant difference was found in socio-economic status between infants with and without follow-up, with a higher SES score in the children that did have follow-up. This finding is similar to findings in previous studies, showing that drop-out was more likely to occur in families with social disadvantages, while preterm children from socially disadvantaged families may have poorer neurodevelopment (3, 32, 43). However, considering the high follow-up rate in this study, limited influence on the presented results is expected.

### Strengths and Limitations

Strengths of our paper included the size of the cohort and the high follow-up rate with most children assessed at three ages. Moreover, the longitudinal nature of this study provides important information reading the developmental course of the children. However, this study has several limitations. First, different tests measuring cognitive performance had to be used, both at different ages to be developmentally appropriate, but also over time in order to use the most recent population norms. Use of different tests intending to measure the same constructs at different ages is inevitable when performing long-term longitudinal studies as development continues at high pace and differentiates strongly during infancy and toddlerhood as well as preschool age (5, 19, 44). In addition, tests need to be re-evaluated and updated over time to allow ecologically valid assessments (e.g., think of the appearance and use of phones in the nineties and zero's, causing the need for revision of the images used in cognitive test). As all tests were standardized however, with a mean of 100, results could be compared. Second, defining NDI based on four determinants (cognitive, neurological, auditory, and visual function) has its limitations to delineate a child's development. Additional domains such as behavioral problems could not be taken into account but may also impair children over time. Third, ideally the GMFCS classification would have been used for classifying the severity of problems in the neurological domain. However, this system was not routinely used in 1990. In order to distinguish between moderate and severe neurological problems, uni- and bi-lateral paresis was used as a proxy for GMFCS 1-2 and GMFCS 3-5, respectively. Last, in this retrospective study, no specific information on interventions was available. Improvement during the trajectories could potentially be the result of adequate interventions after detection of NDI at early age, resulting in improved NDI at early age. Future research may elaborate on the effect of interventions on the individual trajectories.

In conclusion, this study evaluated neurodevelopmental impairment at three different ages up to the age of 8 in very preterm children, next to the course of the longitudinal trajectories in these outcomes. A clear association was found of gestation, gender, and parental education with the severity of NDI at all time points. Although we observed considerable individual variation over time in NDI status, the course of the trajectories in NDI were not associated with gestation, gender, and parental education. These results point to the importance of other (unknown) influences on developmental trajectories. Continued follow-up until school life for extremely preterm born children is essential in order to provide optimal individually focused referrals and care when needed.

## Data Availability Statement

The datasets presented in this article are not readily available because of privacy regulations according to the General Data Protection Regulation (GDPR). Requests to access the datasets should be directed to Pauline E. van Beek, pauline.van.beek@mmc.nl.

## Ethics Statement

The studies involving human participants were reviewed and approved by Medical Ethics Committee Máxima MC. Written informed consent from the participants' legal guardian/next of kin was not required to participate in this study in accordance with the national legislation and the institutional requirements.

## Author Contributions

PB, AB, BV, and PA contributed to the conception of the study. PB, IH, JW, and BV organized the database. PB performed the statistical analysis. PB and IH wrote the first draft of the manuscript. PA was responsible for the financial funding of the project and overall supervision. All authors contributed to the interpretation of the results, critically reviewed the manuscript, and approved the submitted version.

## Conflict of Interest

The authors declare that the research was conducted in the absence of any commercial or financial relationships that could be construed as a potential conflict of interest.

## Abbreviations

NICU: neonatal intensive care unit
GA: gestational age
NDI: neurodevelopmental impairment
BOS 2-30: Bayley Scales 2-30 months, Dutch edition
BSID-II: Bayley Scales of Infant and Toddler Development-II
BSID-III: Bayley Scales of Infant and Toddler Development-III
RAKIT: Revised Amsterdam Child Intelligence Test
RAKIT-2: Revised Amsterdam Child Intelligence Test-II
WPPSI-III-NL: Wechsler Preschool and Primary Scale of Intelligence Scale-III Dutch Edition
WISC-III-NL: Wechsler Intelligence Scale for Children-III Dutch edition.
