# Supplementary material for: Developmental Trajectories in Very Preterm Born Children Up to 8 Years: A Longitudinal Cohort Study
Source: Front Pediatr. 2021 May 10;9:672214. doi: 10.3389/fped.2021.672214 (PMC8143520; doi:10.3389/fped.2021.672214)
Supplement: Supplementary file 1 [file Table_1.DOCX]

**Appendix 1.** Classifications for the separate components of NDI at each follow-up age.

| **2 years** | **Neurology** | **Vision** | **Hearing** | **Cognition** |
| --- | --- | --- | --- | --- |
| None | 614 (88.6) | 590 (85.1) | 620 (89.5) | 495 (71.4) |
| Mild | 32 (4.6) | 94 (13.6) | 68 (9.8) | 89 (12.8) |
| Moderate | 9 (1.3) | 3 (0.4) | 0 (0.0) | 41 (5.9) |
| Severe | 20 (2.9) | 2 (0.3) | 1 (0.1) | 29 (4.2) |
| Unknown | 18 (2.6) | 4 (0.6) | 4 (0.6) | 39 (5.6) |
| **5 years** | **Neurology** | **Vision** | **Hearing** | **Cognition** |
| None | 530 (80.5) | 530 (80.5) | 572 (86.9) | 524 (79.6) |
| Mild | 80 (12.2) | 118 (17.9) | 79 (12.0) | 67 (10.2) |
| Moderate | 15 (2.3) | 5 (0.8) | 2 (0.3) | 27 (4.1) |
| Severe | 19 (2.9) | 0 (0.0) | 0 (0.0) | 1 (0.2) |
| Unknown | 14 (2.1) | 5 (0.8) | 5 (0.8) | 39 (5.9) |
| **8 years** | **Neurology** | **Vision** | **Hearing** | **Cognition** |
| None | 519 (89.6) | 470 (81.2) | 528 (91.2) | 473 (81.7) |
| Mild | 22 (3.8) | 102 (17.6) | 42 (7.3) | 66 (11.4) |
| Moderate | 14 (2.4) | 1 (0.2) | 3 (0.5) | 22 (3.8) |
| Severe | 13 (2.2) | 0 (0.0) | 0 (0.0) | 4 (0.7) |
| Unknown | 11 (1.9) | 6 (1.0) | 6 (1.0) | 14 (2.4) |
